# Pretreatment With BTK Inhibitors Improved the Sensitivity of DLBCL Cells to CAR‐T Cells in a Coculture System by Downregulating the Polarisation of M2 Macrophages

**DOI:** 10.1111/jcmm.71296

**Published:** 2026-07-29

**Authors:** Yao Qi, Xuemei Fan, Jia Wang, Xin Li, Juan Mu, Rui Cui, Qi Deng

**Affiliations:** ^1^ Department of Hematology Tianjin First Central Hospital, School of Medicine and Nankai University Tianjin China; ^2^ The First Central Clinical College of Tianjin Medical University Tianjin China

**Keywords:** Bruton's tyrosine kinase inhibitor, chimeric antigen receptor, diffuse large B‐cell lymphoma cells, macrophages

## Abstract

The tumour microenvironment (TME) of relapsed/refractory (R/R) diffuse large B‐cell lymphoma (DLBCL) patients is associated with resistance of DLBCL cells to CD19 CAR‐T cells. How to improve TME in DLBCL and improve the efficacy of CAR‐T cell therapy remains to be further explored. We observed the sensitivity of HBL‐1/U2932 cells pretreated with BTK inhibitors (BTKi) to CAR‐T cells with flow cytometry (FCM). The effect of pretreatment of BTKi on the polarisation state of alternative activated M2 macrophages was observed with FCM, real‐time PCR and Western blot method. The effect of Notch1 agonist on expressions of Arg‐1 protein, iNOS protein, Notch1 protein and RBP‐J protein in alternative activated M2 macrophages was observed by Western blot method. Then the expression consistency of Notch‐1 and RBP‐J in activated M2 macrophages was observed by siRNA transfection of Notch‐1. The cytotoxicity of CD19 CAR‐T cells on HBL‐1/U2932 cells pretreated with ibrutinib/orelabrutinib was higher than that of HBL‐1/U2932 cells unpretreated with ibrutinib/orelabrutinib. Cytotoxicity of CAR‐T cells to HBL‐1 cells in coculture system with alternative activated M2 macrophages was very low. This drug resistance could be reversed by replacing the M2 macrophages (M2 macrophages after 48 h pretreatment with BTKi) in coculture system. Pretreatment with BTKi could down‐regulate the expression of CD206 and IL‐10 in activated M2 macrophages. And pretreatment with BTKi down‐regulated the expression of Arg‐1 and upregulated the expression of iNOS in activated M2 macrophages. The upregulation polarisation of M2 macrophages by Notch1 agonist could be reversed by BTKi. Expression of RBP‐J protein decreased in alternative activated M2 macrophages by siRNA silencing Notch 1. Pretreatment with BTKi could down‐regulate the polarisation of M2 macrophages and reverse the resistance of DLBCL cells which were cocultured with alternative activated M2 macrophages to CAR‐T cells. This effect might be achieved by downregulating the Notch‐RBP‐J pathway.

## 1 Introduction

Although CD19 chimeric antigen receptor (CAR)‐T‐cell therapy has significantly improved the efficacy and survival of patients with relapsed/refractory (R/R) diffuse large B‐cell lymphoma (DLBCL) [1, 2], many patients with R/RDLBCL do not respond to CD19 CAR‐T‐cell therapy. The mechanism of resistance of DLBCL cells to CD19 CAR‐T cells is not fully understood [3, 4, 5]. Therefore, exploring the causes of drug resistance, optimising CAR‐T‐cell therapy and improving CAR‐T‐cell efficacy are highly clinically important. A study of 51 patients with R/R DLBCL who received CD19 CAR‐T‐cell therapy in a ZUMA‐1 clinical trial revealed that the poor tumour microenvironment (TME) characteristics of patients with R/R DLBCL are related to the resistance of DLBCL cells to CD19 CAR‐T cells [6].

Tumour macrophages are an important part of the TME. M1 macrophages are effector cells induced by pro‐inflammatory cytokines. It could release high levels of pro‐inflammatory cytokines tumour necrosis factor α (TNF‐α) and interferon‐γ (IFN‐γ), thereby exerting anti‐cancer and anti‐infection effects [7, 8]. M2 macrophages promote tumour cell growth and metastasis and induce drug resistance through immunosuppression [9]. The poor survival of patients with DLBCL is related to high‐level polarisation of M2 macrophages [10, 11]. It has also been reported that the polarisation of M2 macrophages inhibits the cytotoxic activity of CD19 CAR‐T cells toward DLBCL cells [12].

Ibrutinib/orelabrutinib is Bruton's tyrosine kinase inhibitor (BTKi) that blocks BTK activation and inhibits B cell receptor (BCR) signalling pathways in B‐cell malignancies [13]. CD19 CAR‐T‐cell therapy has achieved an unexpected and durable response in patients with R/R chronic lymphocytic leukaemia (CLL) who are resistant to ibrutinib [14, 15]. Previous studies on the mechanism by which BTK inhibitors improve the efficacy of CAR‐T cells have focused on increasing the numbers of CD4^+^ and CD8^+^ T cells in patients with CLL by inhibiting the interleukin‐2 (IL‐2)‐induced T‐cell kinase (ITK) pathway. Furthermore, the proliferation and cytotoxic activity of CD19 CAR‐T cells against lymphoma cells could be improved [16]. Long‐term ibrutinib treatment reverses the limited expansion of T cells and plays important roles in the expansion and long‐term maintenance of CAR‐T cells [17, 18]. Ibrutinib can promote the migration of CAR‐T cells to tumours by increasing CD62L expression, which is conducive to the antitumor effect of CAR‐T cells [19]. Our previous study of ibrutinib combined with CD19 CAR‐T cells confirmed that ibrutinib reverses the resistance of B lymphoma cells to CD19 CAR‐T cells in a BALB/c mouse subcutaneous tumour model, but there was no such combined effect in vitro [20]. We reported that ibrutinib promotes CAR‐T‐cell proliferation and downregulates the expression of programmed cell death‐1 in this study. BALB/c mice have a thymus, but the thymus function is incomplete, resulting in weakened immune function and T cell function. Therefore, the T cell activation pathway mediated by the ITK pathway in BALB/c mice is weakened. We hypothesised that the reversal of drug resistance was the result of ibrutinib improving the lymphoma TME. However, how do BTK inhibitors improve the lymphoma TME? This question merits further exploration.

## 2 Materials and Methods

### 2.1 Pharmacologic Agents

The Bruton tyrosine kinase (BTK) inhibitors ibrutinib and orelabrutinib were chosen for our study. Ibrutinib was provided by Xian Janssen Pharmaceutical Co. Ltd. Orelabrutinib was provided by Nuocheng Jianhua Pharmaceutical Company. Ibrutinib and orelabrutinib were dissolved in dimethyl sulfoxide (DMSO) to make a 20 μM stock solution, which was stored at −20°C.

### 2.2 Cell Lines

HBL‐1 and U2932 cells and human THP‐1 macrophages (American Type Culture Collection, ATCC, Manassas, VA, USA) were cultured in RPMI‐1640 medium (Gibco, Thermo Fisher Scientific Inc., Waltham, MA, USA) containing 10% fetal bovine serum (FBS) (Gibco, Thermo Fisher Scientific Inc.) and 50 UI/ml penicillin/streptomycin (Gibco, Life Technologies). Human embryonic kidney 293 (Lenti‐X‐293 T) cells (ATCC) were maintained in Dulbecco's Modified Eagle's Medium (DMEM) (Sigma–Aldrich, USA) supplemented with 10% FBS and 50 UI/mL penicillin/streptomycin.

### 2.3 Inducing Alternatively Activated M2 Macrophages

Human THP‐1 macrophages at the log growth stage were inoculated with 1 × 10^7^ cells per well on 6‐well culture plates and then cultured in serum‐free RPMI 1640 medium supplemented with 100 ng/mL phorbol ester (PMA) and 0.3% BSA for 72 h to induce differentiation. Morphological observation through light microscopy was performed to identify whether the cells differentiated into macrophages. Human THP‐1 macrophages were induced with 20 ng/mL IL‐13 and 20 ng/mL IL‐4 to differentiate into alternatively activated M2 macrophages after 72 h [21].

### 2.4 Generation of Anti‐CD19 CAR‐T Cells

Three volunteers from our research team provided peripheral blood as a source of T cells for the generation of CAR‐T cells. They agreed with the use of their peripheral blood mononuclear cells (PBMCs) for our study. The generation of anti‐CD19 CAR‐T cells was based on previous literature [20].

### 2.5 Construction of a Coculture System of DLBCL Cells and M2 Macrophages

HBL‐1/U2932 cells were cultured in 6‐well culture plates and added to complete culture medium. M2 macrophages induced by THP‐1 macrophages were inoculated into Transwell compartments of microporous polyelectrolyte fibre membranes. The Transwell compartments were placed on a 6‐well culture plate inoculated with DLBCL cells and M2 macrophages and DLBCL cells were cocultured inside and outside the Transwell chamber. This system was used in all coculture systems of macrophages and lymphoma cells for further analysis and detection. A Notch1 agonist (NSC 22423, 2 μmol/L) was added to the coculture system of the Notch1 agonist group.

### 2.6 Pretreatment of Lymphoma Cells and Alternatively Activated M2 Macrophages With BTK Inhibitors

Lymphoma cell lines (HBL‐1/U2932 cells) were cocultured with ibrutinib/orelabrutinib in 2000 μL of medium for 0, 24 and 48 h, with 2 × 10^6^ cells per well. BTK inhibitors were added to the coculture system of lymphoma cell lines on day 0. The concentration of ibrutinib was 1 μmol/L, while the concentration of orelabrutinib was 0.125 μmol/L (the concentrations of ibrutinib/orelabrutinib were determined by the proliferation of HBL‐1/U2932 cells in our preliminary experiment). Similarly, when alternatively activated M2 macrophages were pretreated with BTK inhibitors, changes in the polarisation of the macrophages were observed.

### 2.7 Proliferation Assessment of HBL‐1/U2932 Cells by the CCK‐8 Assay and Apoptosis Assay of M2 Macrophages

To determine the appropriate BTK inhibitor concentration in the coculture system, the effects of different doses of BTK inhibitors on lymphoma cell lines were detected using a Cell Counting Kit‐8 (CCK‐8; Dojindo Molecular Technologies Inc. Kumamoto, Japan). The concentrations of ibrutinib were 0, 1, 2, 4, 6, 8 and 10 μmol/L, whereas the concentrations of orelabrutinib were 0, 0.125, 0.25, 0.5, 1, 2 and 4 μmol/L. The absorbances were measured at 450 nm at 24, 48 and 72 h with an enzyme standard instrument. The experiment was repeated three times.

Apoptosis was determined by staining the inducing alternatively activated M2 macrophages with annexin V and 7‐AAD stains, according to the protocol of the manufacturer (BioLegend, San Diego, CA, USA). Annexin V usually stains early apoptotic cells, whereas double immunostaining with annexin V and 7‐AAD is indicative of late apoptotic/necrotic cells.

### 2.8 Cytotoxicity Assays for Cell Lines

After HBL‐1/U2932 cells/alternatively activated M2 macrophages were pretreated with BTK inhibitors for 48 h, a cytotoxicity assay of CAR‐T cells to HBL‐1/U2932 cells was carried out with an effective target ratio of 1:5 at 24 h/48 h in the absence of supplemented cytokines or BTK inhibitors. An assessment of the cytotoxicity of T cells (from the same source as CAR‐T cells) to HBL‐1/U2932 cells in the control group was also carried out with an effective target ratio of 40:1 at 24 h/48 h. The cells were collected after 24 and 48 h, washed once and resuspended in PBS. All the experiments were repeated three times.

To assess the cytotoxicity of CAR‐T cells to HBL‐1/U2932 cells pretreated with BTK inhibitors, HBL‐1/U2932 cells were stained with 7‐ADD‐H for 30 min at 4°C, washed twice and then quantified by flow cytometry (FCM). The percentage of 7‐ADD‐H (+) cells was represented the percentage of dead HBL‐1/U2932 cells.

In assays of the cytotoxicity of CAR‐T cells to alternatively activated M2 macrophages pretreated with BTK inhibitors, HBL‐1/U2932 cells were fluorescently labelled with 5,6‐carboxyfluorescein diacetatesuccinimidyl ester (CFSE) for 30 min at 4°C and the percentage of dead HBL‐1/U2932 cells was detected by FCM. The cytotoxicity assay of CAR‐T cells against HBL‐1/U2932 cells was performed based on the percentage of dead HBL‐1/U2932 cells. The results of the cytotoxicity assay of CAR‐T cells against HBL‐1/U2932 cells are expressed as follows: the total counts of HBL‐1/U2932 cells and CAR‐T cells at detection time × percentage of dead HBL‐1/U2932 cells at detection time/counts of HBL‐1/U2932 cells at the beginning of coculture.

### 2.9 Single‐Cell RNA‐Seq Analysis

Single M2 macrophages suspension induced by THP‐1 macrophages were prepared from Transwell compartments above. Wash the M2 macrophage suspension with PBS and conduct subsequent experiments immediately. The RNA‐seq library was prepared in accordance with the manufacturer's instructions. To analyse the feature‐barcode matrices generated by 10× Genomics Cell Ranger pipeline through the standard workflow (Seurat v 4.0) in RStudio [22]. M2 macrophage recognition was performed using the reference‐based scRNA‐seq annotation package, SingleR. The differential expression testing functionality of the Seurat package was used to analyse differences in gene expression between two groups of M2 macrophages cocultured with HBL‐1/U2932 cells and ibrutinib/orelabrutinib.

### 2.10 Immunophenotyping and RT‐PCR Detection

The expression levels of CD206 and IL‐10 on macrophages suspended in serum‐free medium were analysed by FCM. For gene expression analysis, total RNA from macrophages was extracted using a NucleoSpin RNA Kit (Macherey‐Nagel), followed by cDNA synthesis with a RevertAid First Strand cDNA Synthesis Kit (Thermo Fisher Scientific) according to the manufacturer's instructions. For gene expression quantification, RQ‐PCR of the template cDNA was performed in triplicate. Real‐time PCR was performed using TaqMan Gene Expression Master Mix and gene expression assays (Applied Biosystems). Arginase‐1 (Arg‐1) and inducible nitric oxide synthase (iNOS) levels were determined relative to those of the reference genes ACTB and GAPDH using the DDCT method. The list of PCR primers is shown in Table 1. All the data were normalized to GAPDH and analysed in triplicate. The experiment was repeated three times.

**TABLE 1:** Sequence detail.

Gene	Forward primer sequence (5′‐3′)	Reverse primer sequence (5′‐3′)
Arg‐1	TCATCTGGGTGGATGCTCACAC	GAGAATCCTGGCACATCGGGAA
iNOS	GCTCTACACCTCCAATGTGACC	CTGCCGAGATTTGAGCCTCATG
GAPDH	CGACGACCCATTCGAACGTCT	CTCTCCGGAATCGAA CCCTGA

### 2.11 Western Blot Analysis

Different groups of macrophages were isolated from the Transwell compartments of microporous polycarbonate fibre membranes. The cells were collected and dissolved in 200 μL of Laemmli buffer (Bio‐Rad, Hercules, CA, USA). Each protein sample was transferred to a PVDF membrane after separation in an SDS–PAGE gel (Bio‐Rad). Anti‐Arg‐1, anti‐iNOS, anti‐Notch1, anti‐transcription factor RBP‐J and anti‐β‐actin antibodies were used as primary antibodies and the corresponding secondary antibodies were used. Total and phosphorylated proteins were detected using an enhanced chemiluminescence detection system. Image J software was used to read the strip grey value. Then the grey value of the target protein was divided by the grey value of the internal parameter and the grey ratio of other groups was divided by the average of the mock grey ratio. The densitometric score of each sample was normalized by β‐actin and was further normalized to the expression of the target protein in each group. The experiment was repeated three times.

### 2.12 siRNA Transfection of Notch‐1

siRNAs targeting specific sequences of Notch‐1 and negative control scrambled siRNA were synthesised by GenePharma (Shanghai, China). The sequences were as follows: Notch1 sense: 5′‐UCGCAUUGACCAUUCAAACUGGUGG‐3′ and antisense: 5′‐CCACCAGUUUGAAUGGUGAAUGCGA‐3′ [23]. Alternatively activated M2 macrophages were seeded into 6‐well plates (1 × 10^5^ cells/well). Then, the siRNA‐Lipofectamine 2000 complex was premixed according to the manufacturer's instructions and added to the 6‐well plates above. We analysed Notch‐1 and RBP‐J expression in alternatively activated M2 macrophages using Western blot analysis 48 h after transfection.

### 2.13 Validation in Vivo in Mice

The establishment of the xenograft tumour model was based on our previous research [20]. Six‐week‐old female CAnN.Cg‐Foxn1nu/CrlVR (BALB/c) mice weighing 20.66 g±1.98 g (*n* = 60; Beijing Vitonlihua Experimental Animal Technology Co. Ltd., Beijing, China) were generated by the subcutaneous injection of lymphoma cells. Upon confirmation of engraftment, the mice were randomised into the ibrutinib group, the CAR‐T‐cell group and the ibrutinib combined with CAR‐T‐cell group (combination group) [20]. The residual tumour tissue of the subcutaneous nodules was taken from each group after 28 days of therapy. Macrophages were extracted from residual tumour tissue with macrophage isolation solution (Sangon Biotech Co. Ltd., Shanghai, China). We confirmed the synergistic effect of ibrutinib and CAR‐T cells in a subcutaneous tumorigenic model [20]. In this study, we validated macrophage expression in mice.

### 2.14 Statistical Analysis

Statistical analysis was performed with GraphPad Prism 7 software. For the comparison between the two groups, the Mann–Whitney *U* (unpaired) test was used and the multiplex *t*‐test was corrected according to the Benjamini‐Hochberg method. For comparisons between more than two groups, the Kruskal–Wallis (unpaired) test was used. All experimental data are presented as the mean ± standard error of the mean (SEM) unless otherwise stated. All *p* values given are two‐tailed values. A *p* value less than 0.05 was considered significant. Sample sizes are provided in the main text.

## 3 Results

### 3.1 Proliferation of HBL‐1/U2932 Cells and Apoptosis of M2 Macrophages Pretreated With/Without Ibrutinib/Orelabrutinib

**FIGURE 1:**
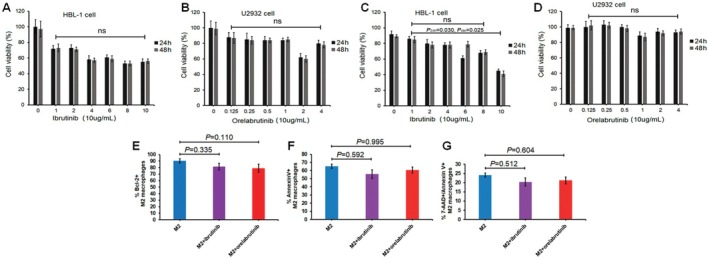
Proliferation of HBL‐1/U2932 cells and apoptosis of M2 macrophages pretreated with/without ibrutinib/orelabrutinib. (A–D) The effects of different doses of ibrutinib/orelabrutinib on the proliferation of HBL‐1/U2932 cells in vitro for 24 h and 48 h. (E–G) No impact of ibrutinib (1 μmol/L)/orelabrutinib (0.125 μmol/L) on the apoptosis of alternatively activated M2 macrophages. (A–G): The statistical tests used were the Mann–Whitney *U* test for two groups and the Kruskal–Wallis test for more than two groups.

Macrophages are one of the important components of the tumour microenvironment and our previous research in mice predicted that BTKi would enhance the efficacy of CAR‐T cells by improving the tumour microenvironment [20]. Therefore, in this study, we first selected macrophages as the research object. The inhibitory effect of ibrutinib/orelabrutinib on the proliferation of HBL‐1/U2932 cells increased with increasing ibrutinib/orelabrutinib concentration at 24 h and 48 h. The inhibitory effect of ibrutinib at a concentration of 10 μmol/L on the proliferation of U2932 cells was greater than that of ibrutinib at a concentration of 1 μmol/L (*p*_24h_ = 0.030 and *p*_48h_ = 0.025). However, there was no difference in the inhibitory effect of ibrutinib/orelabrutinib on the proliferation of HBL‐1/U2932 cells in the other groups and there was no difference in the inhibitory effect between the 24 h and 48 h groups (Figure 1A–D). To explore the effect of BTKi pretreatment on HBL‐1/U2932 cells, we selected the ibrutinib/orelabrutinib concentration that had the least inhibitory effect on the proliferation of HBL‐1/U2932 cells. To minimise the effect of BTKi themselves in inducing the death of DLBCL cells, the concentration of ibrutinib used was 1 μmol/L, whereas the concentration of orelabrutinib used was 0.125 μmol/L in our later study.

We further explored whether ibrutinib/orelabrutinib has an impact on the apoptosis of alternatively activated M2 macrophages. We used BTKi concentrations that did not affect the proliferation of DLBCL cells, 1 μmol/L of ibrutinib and 0.125 μmol/L of orelabrutinib, to observe the effects on the apoptosis of alternatively activated M2 macrophages. Our data show that this concentration of BTKi has no effect on the expression of the anti‐apoptotic protein Bcl‐2 in M2 macrophages. This was evident from the double‐positive immunostaining of the expression of apoptotic protein Bcl‐2^+^ activated M2 macrophages. There was no statistically significant difference in the results of annexin V^+^ single immunostaining and 7‐AAD^+^/Annexin V^+^ double immunostaining in M2 macrophages with or without ibrutinib/orelabrutinib; that is, it did not affect the expression of the anti‐apoptotic protein Bcl‐2 in M2 macrophages. Our results indicated that 1 μmol/L of ibrutinib and 0.125 μmol/L of orelabrutinib did not affect the apoptosis of M2 macrophages (Figure 1E–G).

### 3.2 Cytotoxicity of CAR‐T Cells to HBL‐1/U2932 Cells Pretreated With/Without Ibrutinib/Orelabrutinib

**FIGURE 2:**
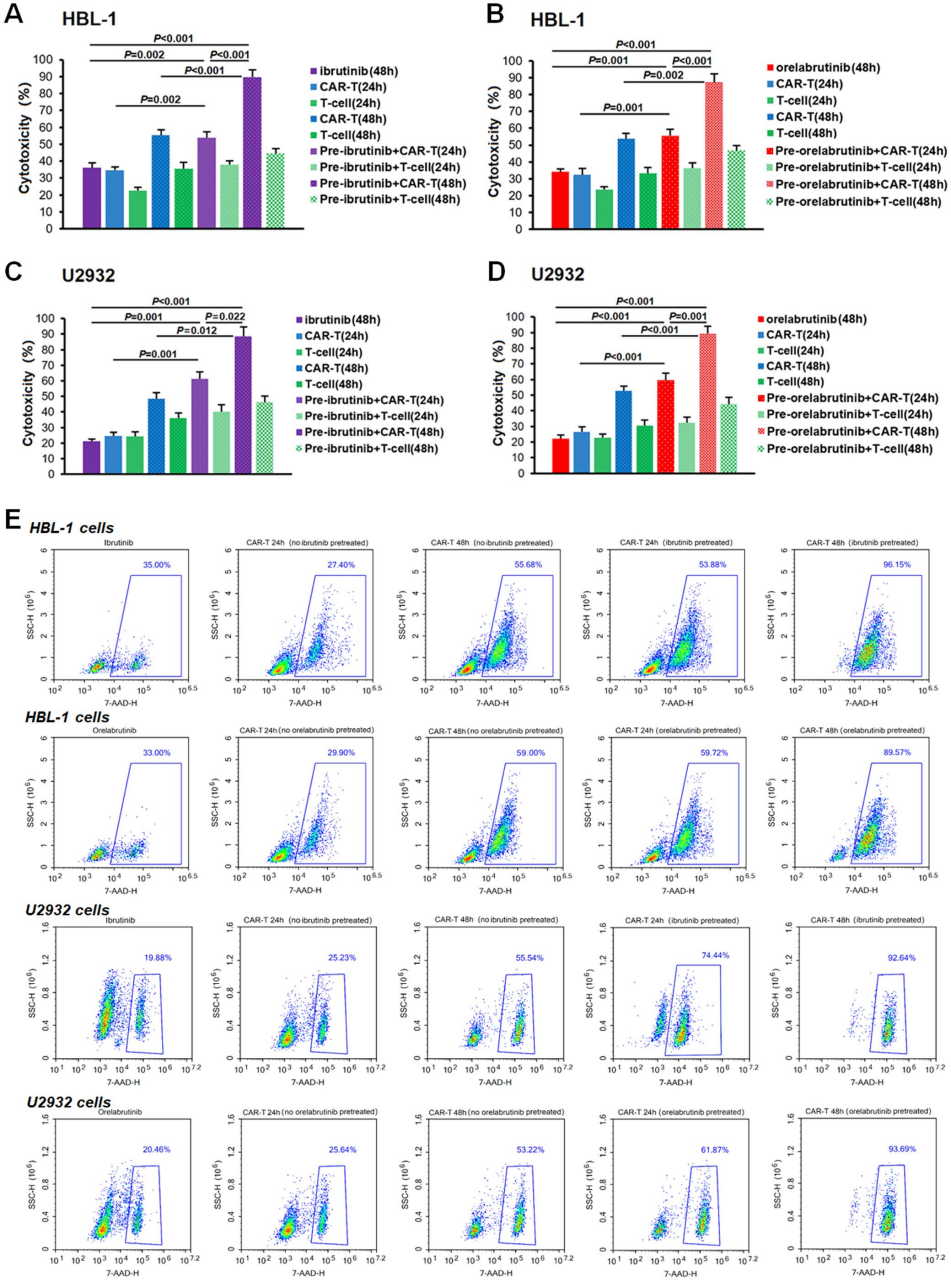
Cytotoxicity of CAR‐T cells to HBL‐1/U2932 cells pretreated with/without ibrutinib/orelabrutinib. (A–D) The cytotoxicity of CAR‐T cells toward HBL‐1/U2932 cells pretreated with ibrutinib (1 μmol/L)/orelabrutinib (0.125 μmol/L) was greater than that toward HBL‐1/U2932 cells not pretreated with ibrutinib/orelabrutinib at 24 h and 48 h. (E) FCM detected the cytotoxicity of CD19 CAR‐T cells against HBL‐1/U2932 cells pretreated with or without BTKi. (A–D): The statistical tests used were the Mann–Whitney *U* test for two groups.

The cytotoxicity of CD19 CAR‐T cells to HBL‐1/U2932 cells pretreated with or without ibrutinib/orelabrutinib was detected by FCM. The cytotoxic activity of ibrutinib/orelabrutinib against HBL‐1/U2932 cells was the result of 48 h coculture. After HBL‐1/U2932 cells were pretreated with ibrutinib/orelabrutinib for 48 h, the cytotoxicity of T cells or CAR‐T cells to HBL‐1/U2932 cells at 24 h and 48 h in the absence of ibrutinib/orelabrutinib is shown in Figure 2A–D. The cytotoxicity of CAR‐T cells toward HBL‐1/U2932 cells pretreated with ibrutinib/orelabrutinib was greater than that of ibrutinib/orelabrutinib group (p*). The cytotoxicity of CAR‐T cells toward HBL‐1/U2932 cells pretreated with ibrutinib/orelabrutinib was greater than that toward HBL‐1/U2932 cells not pretreated with ibrutinib/orelabrutinib (p**). The degree of cytotoxicity was greater in the group pretreated for 48 h than in the group pretreated for 24 h (p***). The proportion of dead HBL‐1/U2932 cells is shown in Figure 2E.

### 3.3 The Expression of the Notch‐1 Signalling Pathway in Alternative Activated M2 Macrophages

**FIGURE 3:**
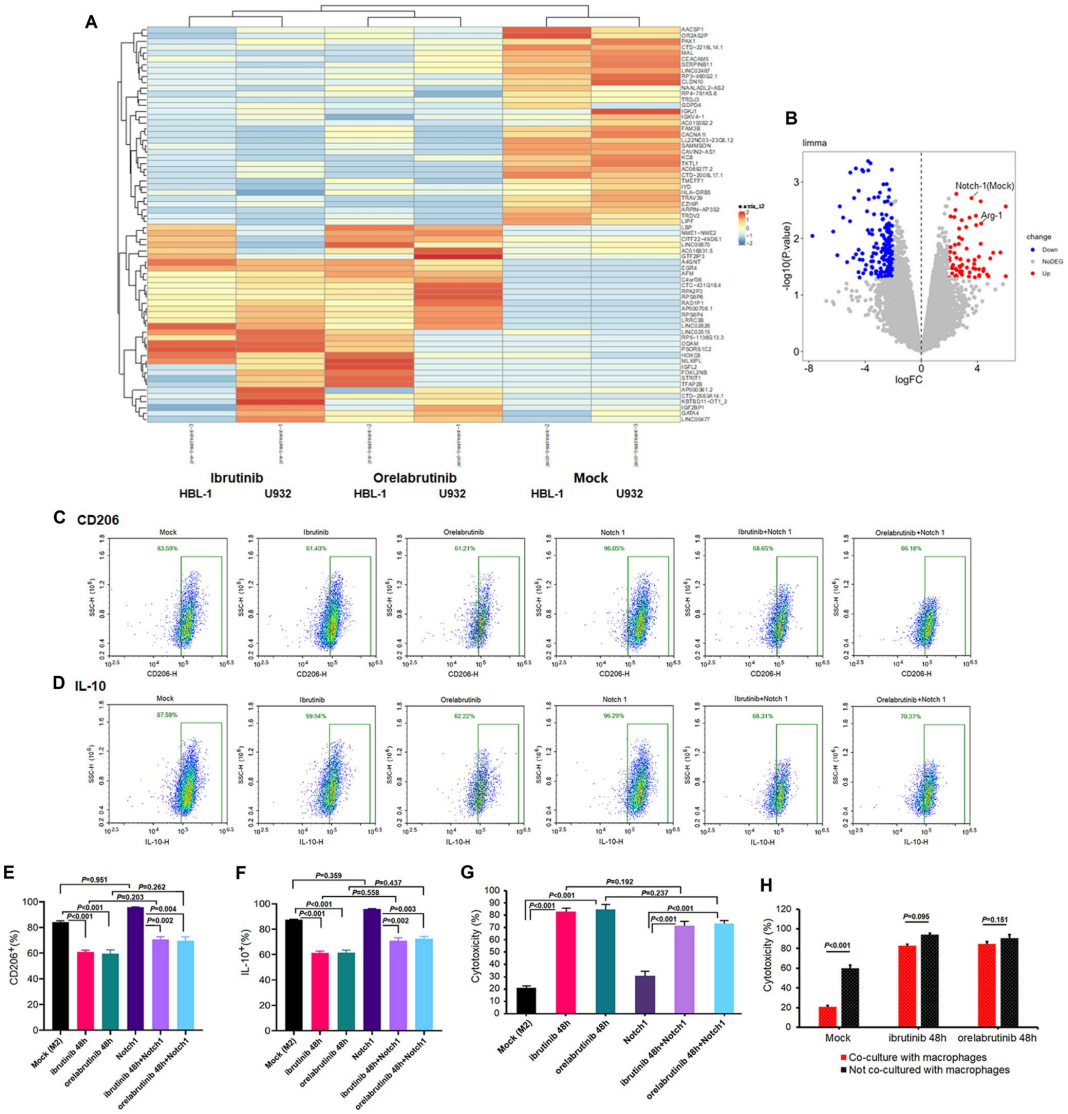
The selection of Notch‐1 signalling pathway in M2 macrophages and the effect of BTKi pretreatment on activated M2 macrophages on CAR‐T cytotoxicity. (A–B) An increased expression of Notch‐1 pathway and Arg‐1 gene in activated M2 macrophages compared with M2 macrophages cocultured with ibrutinib/orelabrutinib. (C–F) The expression of CD206 and IL‐10 in alternative activated M2 macrophages was downregulated by pretreatment with BTKi for 48 h. The upregulation of CD206 and IL‐10 expression by Notch1 agonist could be reversed by BTKi. There was no difference in CD206 and IL‐10 expression between the BTKi group and BTKi + Notch1 agonist group. (G) Cytotoxicity of CAR‐T cells to HBL‐1 cells cocultured with activated M2 macrophages could be reversed by replacing M2 macrophages (pretreatment with BTKi). The drug resistance of HBL‐1 cells to CAR‐T cells induced by Notch1 agonist could be reversed by replacing M2 macrophages (pretreatment with BTKi). (H) The drug resistance of HBL‐1 cells to CAR‐T cells induced by coculturing with activated M2 macrophages could be reversed by replacing M2 macrophages (pretreatment with BTKi). (E–H): The statistical tests used were the Mann–Whitney *U* test for two groups.

To explore the effect of ibrutinib/orelabrutinib on M2 macrophages, we conducted RNA‐seq analysis on it. Volcano plot of gene expression changes represented an increased expression of Notch‐1 signalling pathway and Arg‐1 gene in activated M2 macrophages compared with M2 macrophages cocultured with ibrutinib/orelabrutinib (Figure 3A,B).

### 3.4 Phenotypic Changes in Activated M2 Macrophages by FCM

The immunophenotype of M2 macrophages differentiated from THP‐1 macrophages was verified by FCM. The expression of CD206 in macrophages was 79.14% ± 0.37%, while the expression of IL‐10 in macrophages was 82.73% ± 0.23%. Alternatively activated M2 macrophages were used for subsequent experiments.

The expression levels of CD206 and IL‐10 in alternatively activated M2 macrophages were detected by FCM (Figure 3C,D). In the coculture system of HBL‐1 cells and activated M2 macrophages, the expression levels of CD206 and IL‐10 in activated M2 macrophages increased to 84.34% ± 0.68% and 87.46% ± 0.24%, respectively. The expression levels of CD206 and IL‐10 in the coculture system were downregulated by pretreatment with ibrutinib/orelabrutinib for 48 h. When M2 macrophages were cocultured with a Notch1 agonist (NSC 22423) for 48 h, there was no difference in CD206 or IL‐10 expression between the mock group and the Notch1 agonist group (*p*_*CD206*_ = 0.951 and *p*_*IL‐10*_ = 0.359). Then, the upregulation of CD206 and IL‐10 expression by the Notch1 agonist could be reversed by the addition of ibrutinib (*p*_*CD206*_ = 0.002 and *p*_*IL‐10*_ = 0.002) and orelabrutinib (*p*_*CD206*_ = 0.004 and *p*_*IL‐10*_ = 0.003) to the coculture system for 48 h. However, there was no difference in CD206 or IL‐10 expression between the ibrutinib/orelabrutinib group and the ibrutinib/orelabrutinib + Notch1 agonist group (*p*_*CD206*_ = 0.203 and *p*_*IL‐10*_ = 0.558, *p*_*CD206*_ = 0.262 and *P*_*IL‐10*_ = 0.437) (Figure 3E,F).

### 3.5 Effect of Pretreatment of Activated M2 Macrophages With BTKi Is on the Cytotoxicity of CAR‐T Cells

The cytotoxicity of CAR‐T cells to HBL‐1 cells in the above coculture system with alternatively activated M2 macrophages was very low (Moke). The drug resistance could be reversed by replacing M2 macrophages (M2 macrophages after 48 h of pretreatment with BTKi) in a coculture system (*p*_*ibrutinib*_ < 0.001 and *p*_*orelabrutinib*_ < 0.001). The cytotoxicity of CAR‐T cells to HBL‐1 cells in a coculture system (HBL‐1 cells and alternatively activated M2 macrophages) was decreased following treatment with a Notch1 agonist. The resistance of HBL‐1 cells to CD19 CAR‐T cells induced by a Notch1 agonist was reversed by replacing the M2 macrophages (M2 macrophages after 48 h of pretreatment with BTKi) in a coculture system (*p*_*ibrutinib*_ < 0.001 and *p*_*orelabrutinib*_ < 0.001). There was no difference in the cytotoxicity of CAR‐T cells to HBL‐1 cells between the BTKi pretreatment group and the BTKi pretreatment + Notch1 agonist group (*p*_*ibrutinib*_ = 0.192 and *p*_*orelabrutinib*_ = 0.237) (Figure 3G). The cytotoxicity of CD19 CAR‐T cells to HBL‐1 cells cocultured with alternatively activated M2 macrophages was lower than that of HBL‐1 cells that were not cocultured with activated M2 macrophages (*p* < 0.001). This resistance could be reversed by replacing the M2 macrophages (M2 macrophages after 48 h of pretreatment with BTKi) in a coculture system (*p*_*ibrutinib*_ = 0.095 and *p*_*orelabrutinib*_ = 0.151) (Figure 3H).

### 3.6 Effect of Pretreatment of Activated M2 Macrophages and HBL‐1 Cells With BTKis on the Cytotoxicity of CAR‐T Cells

**FIGURE 4:**
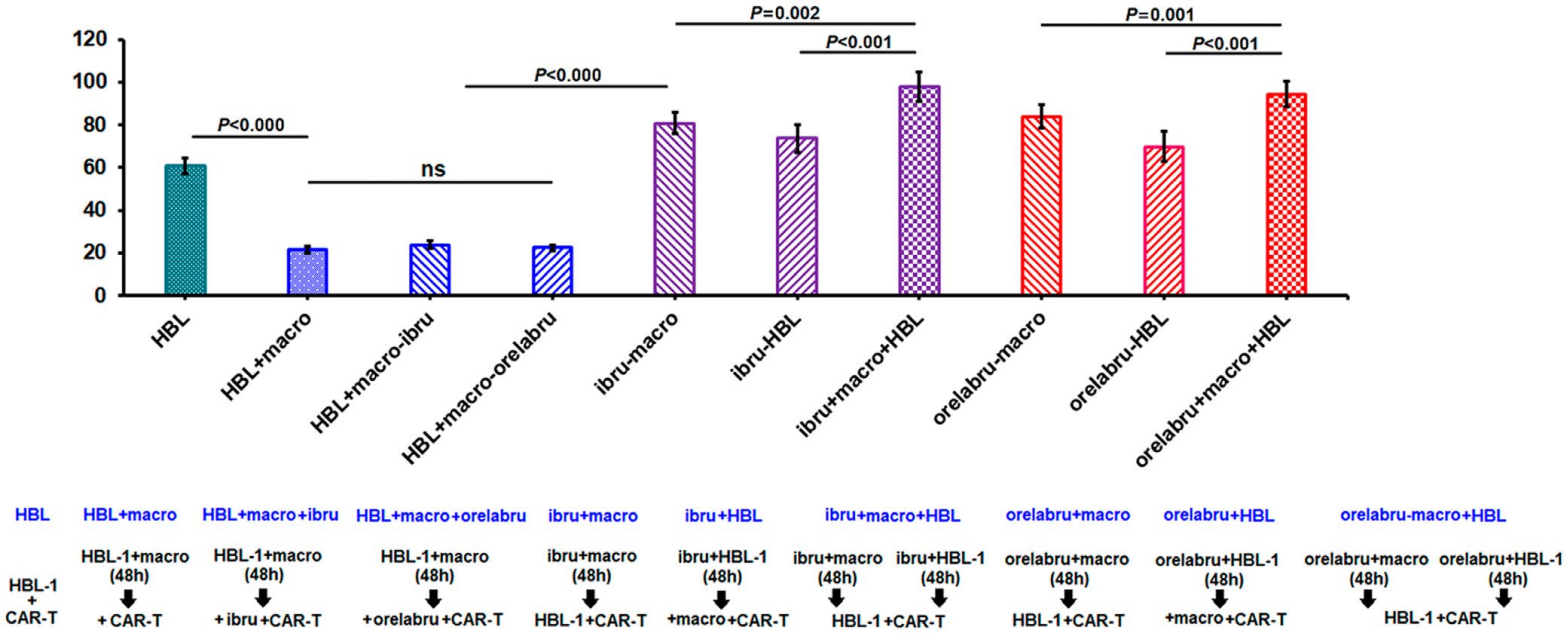
Effect of pretreatment of activated M2 macrophages and HBL‐1 cells with BTKis on the cytotoxicity of CAR‐T cells. After activated M2 macrophages and HBL‐1 cells were pretreated with BTKi respectively, the cytotoxicity of CAR‐T cells to HBL‐1 cells was higher than that of activated M2 macrophages pretreated with BTKi group and higher than that of HBL‐1 cells pretreated with BTKi group. The statistical tests used were the Mann–Whitney *U* test for two groups and the Kruskal–Wallis test for more than two groups.

The cytotoxicity of CAR‐T cells to HBL‐1 cells cocultured with or without alternatively activated M2 macrophages is shown in Figure 4. All the results in this study are the data of HBL‐1 cells/alternatively activated M2 macrophages during BTKi pretreatment or coculture with CAR‐T cells for 48 h. HBL‐1 cells co‐cultured with alternatively activated M2 macrophages for 48 h showed decreased sensitivity to CAR‐T cells (*p* < 0.000). Even if BTK inhibitors are added to the co‐culture system, they cannot increase the sensitivity of HBL‐1 cells to CAR‐T cells. Before the coculture system of alternatively activated M2 macrophages and HBL‐1 cells was constructed, alternatively activated M2 macrophages and HBL‐1 cells were pretreated with BTK inhibitors. After these two cell types were pretreated with ibrutinib or orelabrutinib, the cytotoxicity of CAR‐T cells to HBL‐1 cells was greater than that to alternatively activated M2 macrophages pretreated with ibrutinib or orelabrutinib (*p* = 0.002 and *p* < 0.001) and greater than that to HBL‐1 cells pretreated with ibrutinib or orelabrutinib (*p* = 0.001 and *p* < 0.001) (Figure 4).

### 3.7 Effects of BTKi and a Notch1 Agonist on the Expression of the Arg‐1 and iNOS Genes

**FIGURE 5:**
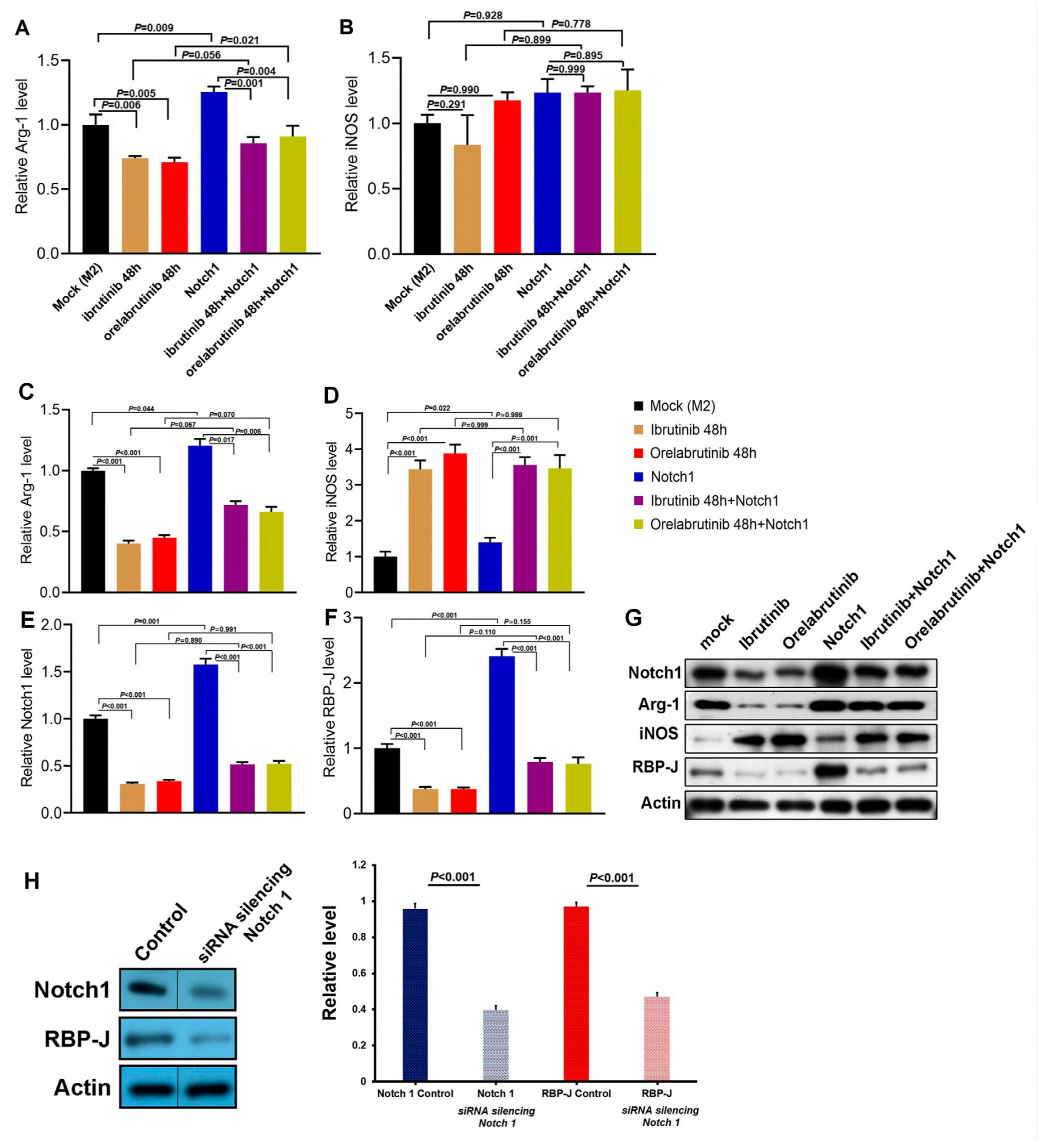
Effects of BTKi and a Notch1 agonist on the expression levels of Arg‐1, iNOS, Notch1 and RBP‐J in activated M2 macrophages. (A) The expression of Arg‐1 gene was down‐regulated by BTKi. The up‐regulation of Arg‐1 gene expression by Notch1 agonist could be reversed by replacing the M2 macrophages (M2 macrophages after 48 h pretreatment with BTKi). There was no difference in Arg‐1 gene expression between the ibrutinib group and ibrutinib + Notch1 agonist group. (B) Pretreatment with BTKi did not affect the expression of iNOS gene. (C) The expression of Arg‐1 protein was down‐regulated by pretreatment with BTKi. The up‐regulation of Arg‐1 protein expression by Notch1 agonist could be reversed by replacing the M2 macrophages (M2 macrophages after 48 h pretreatment with BTKi). There was no difference in expression of Arg‐1 protein in M2 macrophages between BTKi monotherapy group and BTKi pretreatment + Notch1 agonist group. (D) The expression of iNOS protein was up‐regulated by pretreatment with BTKi. The up‐regulation of iNOS protein expression by Notch1 agonist could be reversed by M2 macrophages pretreated with BTKi. There was no difference in expression of iNOS protein between BTKi monotherapy and BTKi pretreatment + Notch1 agonist. (E–F) The changes in Notch1 protein/RBP‐J protein expression in M2 macrophages were consistent with those of Arg‐1 protein expression. (G) The protein levels of the four genes were detected by Western blot. (H) The expression of Notch 1 protein/RBP‐J protein decreased in activated M2 macrophages by siRNA silencing Notch 1. (A–B): The statistical tests used were the Mann–Whitney *U* test for two groups. (C–F): The statistical tests used were the Mann–Whitney *U* test for two groups. (H) The statistical tests used were the Mann–Whitney *U* test for two groups and the Kruskal–Wallis test for more than two groups.

To observe the effects of ibrutinib/orelabrutinib on the expression of M2 macrophages, the expression levels of the Arg‐1 and iNOS genes in the macrophages extracted from the coculture system were quantitatively detected by real‐time PCR. The expression of the Arg‐1 gene in activated M2 macrophages (Mock) was 1.0023 ± 0.0679 and was downregulated by ibrutinib/orelabrutinib pretreatment (*p*_*ibrutinib*_ = 0.006 and *p*_*orelabrutinib*_ = 0.005). The expression of the Arg‐1 gene in M2 macrophages was upregulated by the Notch1 agonist compared with that of the control (Mock) (*p* = 0.009). The increase in Arg‐1 gene expression caused by the Notch1 agonist could be reversed by replacing M2 macrophages (M2 macrophages after 48 h of pretreatment with BTKi) in the coculture system (*p*_*ibrutinib*_ = 0.001 and *p*_*orelabrutinib*_ = 0.004). There was no difference in the expression of the Arg‐1 gene in M2 macrophages between the ibrutinib monotherapy group and the ibrutinib pretreatment + Notch1 agonist group (*p* = 0.056), whereas the expression was greater in the orelabrutinib pretreatment + Notch1 agonist group than in the orelabrutinib monotherapy group (*p* = 0.021) (Figure 5A).

However, in alternatively activated M2 macrophages, the changes in the expression of the iNOS gene were different from those of the Arg‐1 gene. The expression of the iNOS gene in activated M2 macrophages (Mock) was 1.0013 ± 0.0520, though the addition of ibrutinib/orelabrutinib to the coculture system did not affect the expression of the iNOS gene (*p* = 0.291 and *p* = 0.990). The expression of the iNOS gene in M2 macrophages was not upregulated by the Notch1 agonist compared with the control (Mock) (*p* = 0.928). The upregulation of iNOS gene expression by the Notch1 agonist could not be reversed by replacing M2 macrophages (M2 macrophages after 48 h of pretreatment with BTKi) in the coculture system (*p*_*ibrutinib*_ = 0.999 and *p*_*orelabrutinib*_ = 0.895). There was no difference in the expression of the iNOS gene in M2 macrophages between the BTKi monotherapy group and the BTKi pretreatment + Notch1 agonist group (*p*_*ibrutinib*_ = 0.899 and *p*_*orelabrutinib*_ = 0.778) (Figure 5B).

### 3.8 Effects of BTKi and a Notch1 Agonist on the Expression Levels of Arg‐1, iNOS, Notch1 and RBP‐J Proteins in Activated M2 Macrophages

The protein expression levels of Arg‐1 and iNOS in alternatively activated M2 macrophages extracted from the coculture system were quantitatively detected. The protein expression of Arg‐1 in activated M2 macrophages (Mock) was 1.0000 ± 0.0161 and was downregulated by pretreatment with ibrutinib/orelabrutinib for 48 h (*p*_*ibrutinib*_ < 0.001 and *p*_*orelabrutinib*_ < 0.001). Compared with that of the control (Mock), the protein expression of Arg‐1 was increased by the Notch1 agonist (*p* = 0.044). The upregulation of Arg‐1 protein expression by the Notch1 agonist could not be reversed by replacing the M2 macrophages (M2 macrophages after 48 h of pretreatment with BTKi) in the coculture system (*p*_*ibrutinib*_ = 0.017 and *p*_*orelabrutinib*_ = 0.006). There was no difference in the protein expression of Arg‐1 in M2 macrophages between the BTKi monotherapy group and the BTKi pretreatment + Notch1 agonist group (*p*_*ibrutinib*_ = 0.067 and *p*_*orelabrutinib*_ = 0.070) (Figure 5C).

However, the protein expression of iNOS protein in alternatively activated M2 macrophages was opposite that of the Arg‐1 gene. The protein expression of iNOS in activated M2 macrophages was 1.0000 ± 0.1129 and was upregulated by pretreatment with ibrutinib/orelabrutinib (*p*_*ibrutinib*_ < 0.001 and *p*_*orelabrutinib*_ < 0.001). The protein expression of iNOS was downregulated by the Notch1 agonist (*p* = 0.022). The downregulation of iNOS protein expression by the Notch1 agonist could not be reversed by replacing M2 macrophages in the coculture system (*p*_*ibrutinib*_ < 0.001 and *p*_*orelabrutinib*_ = 0.001). There was no difference in the protein expression of iNOS in M2 macrophages between the BTKi monotherapy group and the BTKi pretreatment + Notch1 agonist group (*p*_*ibrutinib*_ = 0.999 and *p*_*orelabrutinib*_ = 0.999) (Figure 5D).

The protein expression of Notch1 in alternatively activated M2 macrophages extracted from the coculture system was quantitatively detected. The expression of the Notch1 protein in activated M2 macrophages was 1.0000 ± 0.0295 and was downregulated by pretreatment with ibrutinib/orelabrutinib (*p*_*ibrutinib*_ < 0.001 and *p*_*orelabrutinib*_ < 0.001). The Notch1 protein in alternatively activated M2 macrophages was upregulated by the Notch1 agonist (*p* = 0.001). The upregulation of Notch1 protein expression by the Notch1 agonist could not be reversed by replacing M2 macrophages in the coculture system (*p*_*ibrutinib*_ < 0.001 and *p*_*orelabrutinib*_ < 0.001). There was no difference in the protein expression of Notch1 in M2 macrophages between the BTKi monotherapy group and the BTKi pretreatment + Notch1 agonist group (*p*_*ibrutinib*_ = 0.890 and *p*_*orelabrutinib*_ = 0.991) (Figure 5E).

The expression of the RBP‐J protein in the alternatively activated M2 macrophages extracted from the coculture system was quantitatively detected. The RBP‐J protein level in activated M2 macrophages was 1.0000 ± 0.0540 and was downregulated by pretreatment with ibrutinib/orelabrutinib (*p*_*ibrutinib*_ < 0.001 and *p*_*orelabrutinib*_ < 0.001). The RBP‐J protein in alternatively activated M2 macrophages was upregulated by the Notch1 agonist (*p* < 0.001). The upregulation of RBP‐J protein expression by the Notch1 agonist could not be reversed by replacing M2 macrophages in the coculture system (*p*_*ibrutinib*_ < 0.001 and *p*_*orelabrutinib*_ < 0.001). There was no difference in the protein expression of RBP‐J in M2 macrophages between the BTKi monotherapy group and the BTKi pretreatment + Notch1 agonist group (*p*_*ibrutinib*_ = 0.110 and *p*_*orelabrutinib*_ = 0.155) (Figure 5F).

Western blotting was performed to evaluate the expression levels of the iNOS, Arg‐1, Notch1 and RBP‐J proteins in alternatively activated M2 macrophages (Figure 5G).

### 3.9 RBP‐J Has a Notch1 Binding Site and Is Regulated by Notch1

RBP‐J has a Notch1 binding site and is regulated by Notch1. The expression of the Notch 1 protein decreased in alternatively activated M2 macrophages in response to siRNA‐mediated silencing of Notch1 (*p* < 0.001). Moreover, the protein expression of RBP‐J decreased in alternatively activated M2 macrophages in response to siRNA‐mediated silencing of Notch 1 (*p* < 0.001) (Figure 5H).

### 3.10 The Effect of Ibrutinib on Macrophages in Mice

**FIGURE 6:**
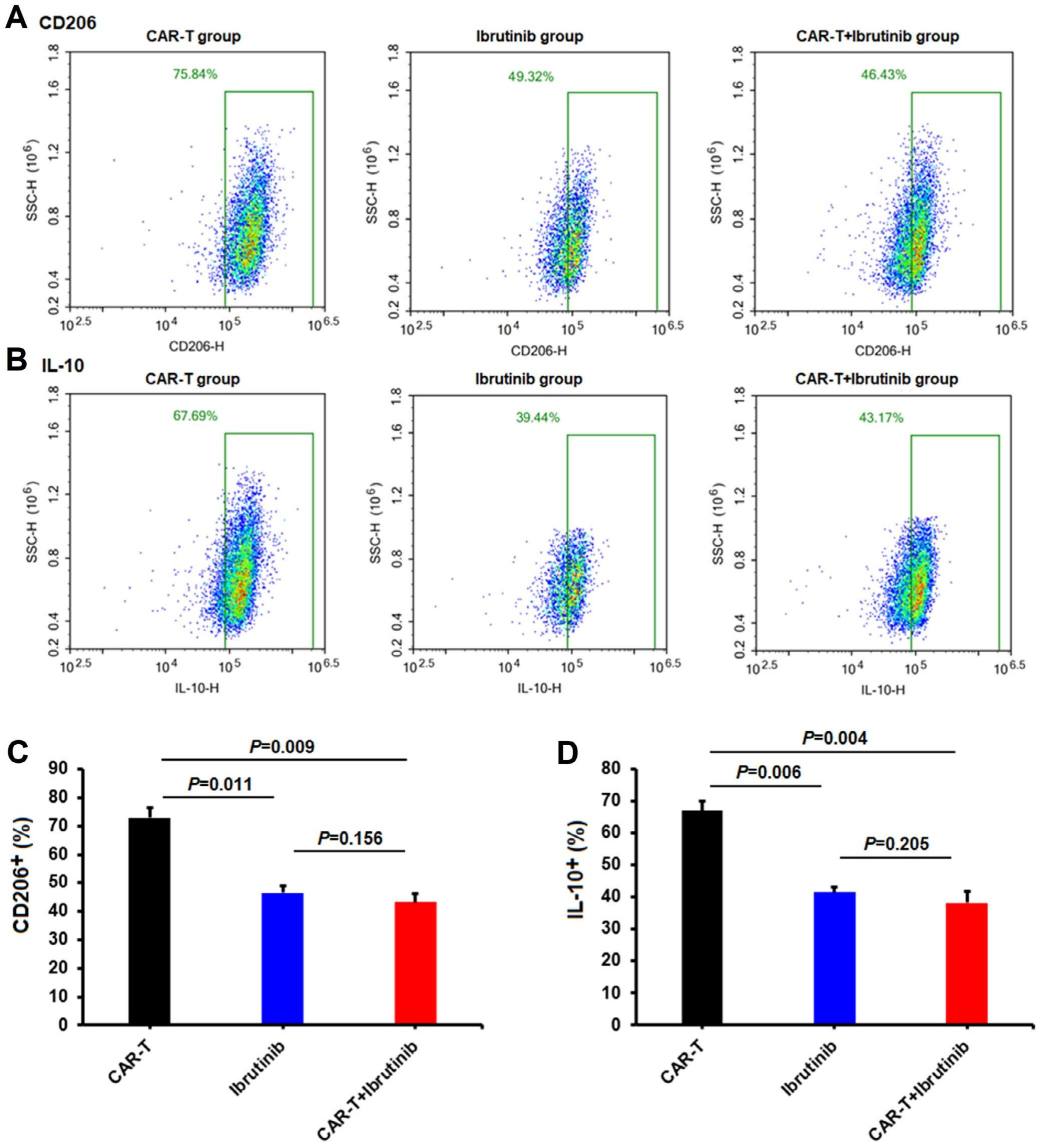
The influence of ibrutinib on expression of macrophages in mice. (A–D) CD206 and IL‐10 expression in macrophages from residual tumour tissue in mice were down‐regulated in the ibrutinib group and CAR‐T+ ibrutinib group. (C–D): The statistical tests used were the Mann–Whitney *U* test for two groups and the Kruskal–Wallis test for more than two groups.

**FIGURE 7:**
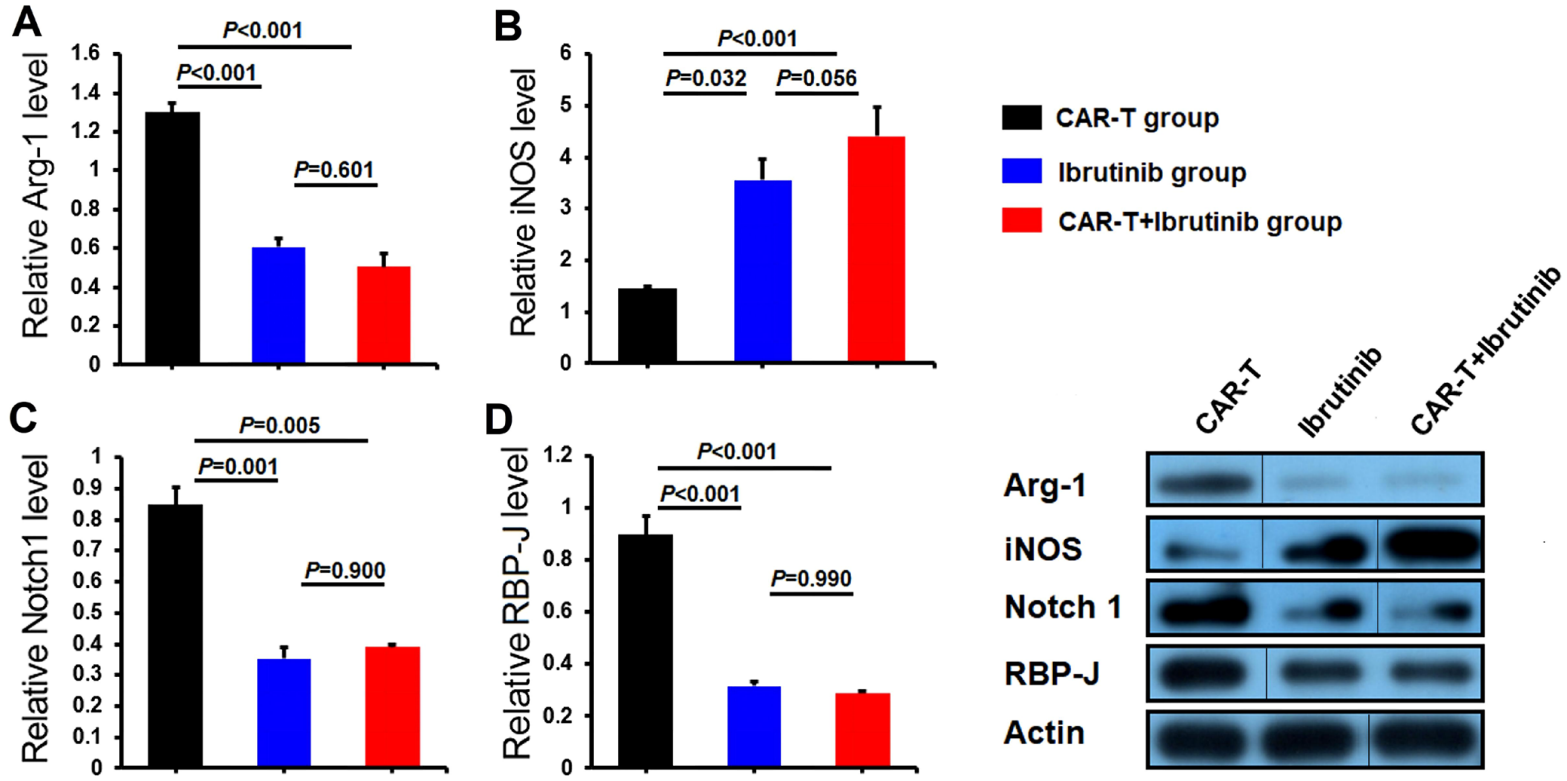
Expression of Arg‐1, iNOS, Notch1 and RBP‐J proteins in macrophages in mice. (A–D) Arg‐1 protein, Notch1 protein and RBP‐J protein expressions were down‐regulated in the ibrutinib group and CAR‐T+ ibrutinib group in mice. The iNOS protein was up‐regulated in the ibrutinib group and CAR‐T+ ibrutinib group in mice. (A–D): The statistical tests used were the Mann–Whitney *U* test for two groups and the Kruskal–Wallis test for more than two groups.

Macrophages from lymphoma tissue in mice in our previous study were extracted for the following study [20]. CD206 and IL‐10 expression levels in macrophages from residual tumour tissue were lower in the ibrutinib group (*p* = 0.011 and *p* = 0.006) and the CAR‐T + ibrutinib group (*p* = 0.009 and *p* = 0.004) than in the CAR‐T group. There was no difference in CD206 or IL‐10 expression in macrophages between the ibrutinib group and the CAR‐T + ibrutinib group (Figure 6A–D). The expression levels of the Arg‐1, Notch1 and RBP‐J proteins in residual tumour tissue were downregulated in the ibrutinib group (*p* < 0.001, *p* = 0.001 and *p* < 0.001) and the CAR‐T + ibrutinib group (*p* < 0.001, *p* = 0.005 and *p* < 0.001) compared with the CAR‐T group. The expression of the iNOS protein was upregulated in both the ibrutinib group (*p* = 0.032) and the CAR‐T + ibrutinib group (*p* < 0.001) (Figure 7A–D). However, there was no difference in protein expression in macrophages between the ibrutinib group and the CAR‐T‐cell + ibrutinib group.

## 4 Discussion

In our clinical trial of CD19 CAR‐T‐cell therapy (ChiCTR‐ONN‐16009862) for R/R B‐cell lymphoma, after patients with R/R B‐cell lymphoma failed to respond to the first CD19 CAR‐T‐cell therapy, the resistance of CAR‐T cells was reversed after 7–16 months of ibrutinib salvage therapy. They obtained very satisfactory efficacy in the second round of CD19 CAR‐T‐cell therapy [24]. In our further exploration, we found that in BALB/c mice with subcutaneous tumour formation of B lymphoma cell lines, ibrutinib reversed the resistance of lymphoma cells to CAR‐T cells and improved efficacy. Moreover, we observed significant expansion of CAR‐T cells in the combined group of lymphoma BALB/c mice [13]. What is the mechanism by which BTK inhibitors reverse the resistance of CAR‐T cells and improve their efficacy? The tumour tissue of subcutaneous tumorigenic mice was considered to have formed a local mass with a local TME. The TME has been shown to play important roles in the drug resistance and progression of DLBCL [10, 11]. The TME promotes drug resistance and progression by inhibiting effector T‐cell activity and recruiting immunosuppressive cells such as macrophages [25, 26, 27]. A previous study reported the immunomodulatory effect of BTK inhibitors on macrophages, which is realised by effectively downregulating the secretion of the homeostatic chemokines CXCL12, CXCL13 and CCL19, as well as the angiogenic cytokine VEGF [28]. BTK inhibitors could be used to target TAMs in the tumour microenvironment and modify the immune landscape. This finding is consistent with our viewpoint, but the specific mechanism needs to be explored further. In this study, we preliminarily explored improvements in the TME and the regulation of macrophage M2 polarisation by BTK inhibitors. Activation of the Notch pathway leads to the polarisation of tumour‐associated macrophages into the M2 phenotype and promotes tumour cell proliferation [29]. The transcription factor RBP‐J is a core participant in Notch signalling and the Notch signalling pathway mediates the activation of macrophages through the transcription factor RBP‐J [30].

First, we analysed the effects of different doses of BTK inhibitors on DLBCL cells (HBL‐1/U2932 cells) and the sensitivity of DLBCL cells to CAR‐T cells after pretreatment with BTK inhibitors. We found that HBL‐1/U2932 cells were more sensitive to CAR‐T cells after pretreatment with ibrutinib/orelabrutinib. Moreover, HBL‐1/U2932 cells pretreated with ibrutinib/orelabrutinib for 48 h were more sensitive to CAR‐T cells than were those pretreated for 24 h. However, in our previous studies, we did not find a synergistic effect of simultaneous coculture of CAR‐T cells and BTK inhibitors in vitro [13]. Based on these results, we further studied the effects of pretreatment with BTK inhibitors on alternatively activated M2 macrophages.

We successfully constructed alternatively activated M2 macrophages from human THP‐1 macrophages. After coculture with HBL‐1 cells, the expression levels of CD206 and IL‐10 in activated M2 macrophages were upregulated. However, the expression levels of CD206 and IL‐10 were downregulated by ibrutinib/orelabrutinib. Interestingly, the upregulation of CD206 and IL‐10 expression by the Notch1 agonist could be reversed by pretreatment with ibrutinib/orelabrutinib. Meanwhile, the changes in the expression of Arg‐1 gene and protein we obtained also confirmed this result. The change in the expression of iNOS protein also proves our point, but the change in the expression of iNOS gene is inconsistent with that of iNOS protein. We repeated the experiments many times and they were indeed inconsistent. We analysed that it could also be related to the instability of alternatively activated M2 macrophages. We further explored this result in the subsequent research. Moreover, the sensitivity of HBL‐1 cells cocultured with alternatively activated M2 macrophages from each group to CAR‐T cells changed accordingly. We further compared the sensitivity of HBL‐1 cells that were cocultured with or without alternatively activated M2 macrophages to CAR‐T cells. HBL‐1 cells cocultured with alternatively activated M2 macrophages presented significantly decreased sensitivity to CAR‐T cells, but this effect could be reversed by the pretreatment of M2 macrophages with BTK inhibitors. We conclude that pretreatment with BTK inhibitors decreases the polarisation of M2 macrophages and reverses the resistance of HBL‐1 cells to CAR‐T cells induced by alternatively activated M2 macrophages.

In our mechanistic exploration, we also revealed that a Notch1 agonist increased the polarisation of M2 macrophages and further induced resistance in HBL‐1 cells in the coculture system to CAR‐T cells. The activation of the Notch1 receptor in malignancies was first reported in T‐cell acute lymphoblastic leukaemia [31]. Many subsequent studies have shown that Notch1 receptor activation occurs in a variety of tumours, including CLL [32], mantle cell lymphoma [33] and DLBCL [34]. Patients with Notch1 mutations are often associated with a greater risk of disease progression, drug resistance and poorer clinical outcomes [35, 36]. However, whether the Notch1 pathway regulates the polarisation of M2 macrophages remains unclear. It has been reported that Notch1 signalling can affect macrophage subtypes and function [37, 38]. When Notch1 signalling is blocked, macrophages are inhibited from differentiating into the M2 macrophage type [39, 40]. It has also been reported that activation of the Notch‐RBP‐J pathway promotes polarisation of M2‐type macrophages [41]. The Notch signalling pathway regulates cell differentiation and proliferation. Then, the Notch intracellular domain (NICD) translocates to the nucleus and binds to the DNA‐binding protein RBP‐J [41]. The most definitive function of Notch signalling is to regulate the function of lymphocytes [42] and it has been reported that the Notch pathway can regulate myeloid cell function [43, 44, 45]. However, the mechanism of the effect of the Notch‐RBP‐J pathway on the polarisation of macrophages remains unclear.

In our study, we found that pretreatment with ibrutinib/orelabrutinib downregulated the polarisation of M2 macrophages in each group of coculture systems. The number of macrophages extracted from the coculture system with HBL‐1 cells was detected. The polarisation of M2 macrophages was downregulated by pretreatment with ibrutinib/orelabrutinib but was upregulated by pretreatment with a Notch1 agonist. The increased polarisation of M2 macrophages induced by a Notch1 agonist could be reversed by pretreatment with ibrutinib/orelabrutinib. Interestingly, the resistance of M2 macrophage‐induced HBL‐1 cells to CAR‐T cells could be reversed by ibrutinib/orelabrutinib pretreatment of M2 macrophages.

In our further exploration of the mechanism by which pretreatment with BTK inhibitors downregulates the polarisation of M2 macrophages, we found that the expression of the RBP‐J protein was consistent with that of the Notch1 protein. We verified the consistency of Notch1 and RBP‐J expression in activated M2 macrophages by the siRNA‐mediated transfection of Notch1.

Therefore, we first found that HBL‐1/U2932 cells were more sensitive to CAR‐T cells after being pretreated with BTK inhibitors. Secondly, we found that activation of the Notch‐RBP‐J pathway promoted the polarisation of M2 macrophages. Moreover, we further demonstrated that pretreatment with BTK inhibitors downregulated the Notch‐RBP‐J pathway in M2 macrophages and subsequently reversed the resistance of DLBCL cells cocultured with reactivated M2 macrophages to CAR‐T cells. We also demonstrated our results in a mouse model of lymphoma. M2 macrophages are an important part of the TME of R/R DLBCL and our study might prove that BTK inhibitors could improve the TME and further reverse the resistance of DLBCL cells to CAR‐T cells.

## Author Contributions

**Xuemei Fan:** formal analysis, investigation, visualization, writing – review and editing. **Qi Deng:** conceptualization, funding acquisition, project administration, resources, supervision, writing – review and editing, validation. **Xin Li:** resources, supervision, software, writing – review and editing. **Jia Wang:** data curation, formal analysis, methodology, resources, software, visualization, writing – review and editing. **Juan Mu:** project administration, methodology, writing – review and editing. **Yao Qi:** conceptualization, funding acquisition, data curation, formal analysis, investigation, methodology, project administration, writing – original draft, writing – review and editing. **Rui Cui:** conceptualization, project administration, resources, supervision, writing – review and editing.

## Funding

This work was supported by the Tianjin Health Research Project (TJWJ2023ZD003) and the Chinese Society of Clinical Oncology Beijing Xisike Clinical Oncology Research Foundation (Y‐2024AZ(BTK)QN‐0005 and Y‐2022YMJN/MS‐0001).

## Conflicts of Interest

The authors declare no conflicts of interest.

## Data Availability

The data that support the findings of this study are available from the corresponding author upon reasonable request.
